# Ensemble Classifier Based on Interval Modeling for Microarray Datasets

**DOI:** 10.3390/e26030240

**Published:** 2024-03-08

**Authors:** Urszula Bentkowska, Wojciech Gałka, Marcin Mrukowicz, Aleksander Wojtowicz

**Affiliations:** Institute of Computer Science, University of Rzeszów, 35-310 Rzeszów, Poland; wgalka@ur.edu.pl (W.G.); mmrukowicz@ur.edu.pl (M.M.); alwojtowicz@ur.edu.pl (A.W.)

**Keywords:** multi-class classification, microarrays, ensemble classification, entropy, cross-entropy, aggregation functions, interval modeling

## Abstract

The purpose of the study is to propose a multi-class ensemble classifier using interval modeling dedicated to microarray datasets. An approach of creating the uncertainty intervals for the single prediction values of constituent classifiers and then aggregating the obtained intervals with the use of interval-valued aggregation functions is used. The proposed heterogeneous classification employs Random Forest, Support Vector Machines, and Multilayer Perceptron as component classifiers, utilizing cross-entropy to select the optimal classifier. Moreover, orders for intervals are applied to determine the decision class of an object. The applied interval-valued aggregation functions are tested in terms of optimizing the performance of the considered ensemble classifier. The proposed model’s quality, superior to other well-known and component classifiers, is validated through comparison, demonstrating the efficacy of cross-entropy in ensemble model construction.

## 1. Introduction

Classification algorithms are essential data mining techniques for real-world applications [1]. They use a model based on the dataset’s contents to classify new objects or understand the existing class distribution [2]. Classification has many applications, such as finding patterns in financial markets, automatically labelling large image collections, and supporting bank loan decisions [3,4]. It is also widely used in medical systems [5]. Classification is a supervised data mining method that involves model building, testing, and predicting unknown values.

A classifier is a computational model or method that assigns input data examples to predefined classes. This supervised learning method uses labelled training data to teach the classifier how to predict or judge the class labels of future or unseen instances. A classifier can be defined as a function or mapping that assigns a feature vector representing an input instance x to one of the specified classes. A classifier can be expressed mathematically as f:X→Y, where *X* is the feature space or input domain and *Y* is the set of possible class labels or output domain. The function *f* maps an input instance *x* from *X* to its corresponding class label *y* in *Y*.

Multi-class classification is a type of classification that deals with many classes, but it seems it is less studied than binary classification, which only has two classes [6]. Unlike binary classification, which separates normal and abnormal cases [7], multi-class classification assigns examples to one of the known classes. However, multi-class problems can have a multitude of attributes that influence the classification outcome, making the task more complex and challenging. It is crucial to consider these attributes during model development to ensure accurate and reliable results [8].

This contribution aims to introduce a new method of multi-class classification using interval modeling, which can handle the challenges of multi-class problems in the case of a large number of attributes. In this work, we concentrated on microarrays (used to measure the expression levels of large numbers of genes simultaneously or to genotype multiple regions of a genome) which are typical examples of high-dimensional datasets. However, the proposed models may be applied to other high-dimensional datasets. Technical solutions regarding the implementation of the algorithms allow to test the models on high-dimensional datasets. However, the quality of performance of the proposed models in the case of datasets other than microarrays are unpredictable.

The proposed ensemble classifier is a heterogeneous type, since it is based on diverse classification algorithms. As was stressed in [9], known strong classifiers are tree-based ones (e.g., Random Forest [10]), Support Vector Machines [11,12], or Multilayer Perceptrons/Deep Neural Networks [13]. In that paper, the authors presented the results of a test performed on over 200 datasets and claimed that instead of optimizing the mentioned single models, it is better to build an ensemble classifier of the mentioned models. The comparative studies of classifiers give some indication which classifier to apply, e.g., Ref. [14]—it is a suggestion which classifier is better on average over standard problems, but it seems that a definitive answer is not possible to be reached. In the situation of a lack of domain knowledge, it is reasonable to ensemble classifiers from different families rather than optimizing a specific type. In our approach as a base classifier, the simple and well-known k Nearest Neighbors (k-NN) classifier [15] was also applied, which proved to work well in many applications. Scalability and interpretability are also the reason to use these simpler classifiers. Since the proposed approach of classification is a multi-class, we also compared our results with well-known decomposition techniques, such as one-versus-one (OVO) and one-versus-rest (OVR), which reduce the multi-class problem to multiple binary problems. The main reason of applying these methods is the fact that decomposition strategies prove to be profitable even when they are not required. This result also holds for k-NN [16]. A comparison has since been made with the basic version of this algorithm (directly suitable for the multi-class case) as well as with the corresponding decomposiotion models. As a base classifier for decomposition, the k-NN classifier is applied. Moreover, Bagging [17]—a well-known ensemble model—was considered to compare the results obtained by the proposed models.

The approach presented employs interval modeling to address uncertainty in the classification process [18,19,20]. Intervals are generated from the predictions of component classifiers and then aggregated using interval-valued aggregation functions. The presented method assigns the decision class of an object by using interval order [21,22]. The paper also investigates how the choice of different interval-valued aggregation functions can affect the classifier performance. We conduct a comparative analysis of three ensemble classifier methodologies. Each of these methodologies employs interval modeling, with two of them additionally incorporating cross-entropy.

The Proposed Model trains a set of models on a dataset. Each model within a given group is individually trained on these data. This process is repeated for each group of models, resulting in a set of trained models ready for predictions. The Proposed Entropy Model differs from the Proposed Model in several ways. It introduces a cross-validation process using a repeated stratified K-fold, which splits the training data into different folds. For each model, it calculates the cross-entropy loss for each fold and stores these losses, then it selects the models based on their mean losses. Models with losses less than or equal to the average loss are chosen, while others are removed. The Proposed Entropy Groups Model introduces additional steps compared to the Proposed Entropy Model. It calculates the mean cross-entropy loss (20) for each group of models. It then filters the groups based on their mean losses, keeping only those with a mean loss less than or equal to the average. If fewer than two groups remain after this filtering, it keeps the two groups with the lowest mean losses.

The obtained results show that interval modeling may be successfully applied in multi-class ensemble classification, outperforming the single classifiers’ performance. Furthermore, the performance of the evaluated ensemble classifier may be significantly enhanced by employing cross-entropy as a criterion to select the most effective component classifiers, which was proved using statistical tests.

The manuscript is organized as follows. In Section 2, a brief literature review is provided concerning multi-class classification methods for high-dimensional datasets. Section 3 provides basic concepts related to interval calculus and interval-valued aggregation functions. Section 4 presents characteristic of microarray datasets applied in the experiments. Section 5 and Section 6 give information about the applied methodology and proposed models. In Section 7, the results of the experiments are presented, while in Section 8, a discussion on these results is provided.

## 2. Literature Review

The existing literature [23,24,25] explores various techniques for performing multi-class classification tasks with many conditional attributes. One of the studies [23] demonstrated that the support vector machine is an effective method for classifying multi-class breast cancer data with high dimensionality. The study compared the performance of Support Vector Machine with other methods, such as Naive Bayes, Random Forest, and multinomial Logistic Regression, and showed that the latter methods are prone to overfitting in this scenario. Another study [24] developed a new learning algorithm called Latent-ISVM for achieving accurate multi-class image classification with very large datasets. The algorithm uses a latent variable model to capture the underlying structure of the images and a kernel function to map the images to a high-dimensional feature space. The study claims that Latent-ISVM can handle complex and diverse image data better than other methods. A third study [25] presented a novel method called SEGEP (Sigmoid-based Ensemble Gene Expression Programming) for multi-class classification with high-dimensional and low-sample-size (HDLSS) data. The method integrates a flexible probability representation, an effective data splitting mechanism, and a unique sampling strategy to deal with output conflicts and improve binary classifiers. The study conducts experiments on several HDLSS datasets and shows that SEGEP outperforms other genetic programming methods for multi-class classification in this setting.

As can be noticed, diverse approaches have been taken to cope with the problem of multi-class classification. In our approach, we intended to use interval modeling, which was successfully applied in diverse areas of classification (cf. [18,19,20]). To our knowledge, this is one of the first contributions in the field of multi-class classification which employs interval modeling. The Interval-Valued version of the decomposition OvO (one-versus-one) approach, proposed as an alternative strategy in [26], is a notable academic contribution in the field of multi-class classification. In the proposed models, we do not use decomposition techniques.

Entropy is an important concept which may have diverse applications; one of them is using it in ensemble classification (cf. [27,28,29]). In [30], information entropy was used to measure the diversity of component classifiers. A novel algorithm, which utilizes the information entropy theory to assess classification outcomes, was introduced according to [31]. It uses ensemble classification techniques, and the weight of each classifier is decided through the entropy of the result produced by an ensemble classifiers system. Weighted entropy was used in an ensemble model, which aims to automatically manage the strengths and weaknesses of each of its separate models [32]. Entropy Convolutional Neural Network was used to estimate Ensemble Deep Learning [33]. The proposed method in [34] Entropy-based Hybrid Sampling Ensemble Learning (EHSEL) is used for imbalanced datasets. The method takes the distributions of the training data into consideration by the information entropy and as a result distinguishing the important samples in the undersampling procedure. In addition, the EHSEL is applied to three different kinds of basic classifiers to validate its robustness.

In this contribution, we propose and compare three ensemble classification models (based on interval modeling). In two out of three proposed models, cross-entropy (20) (unlike the previously discussed works, a different type of entropy measure) is employed to choose the most promising component models in the ensemble classifier. It will be shown that the models with cross-entropy involved significantly outperform the model without cross-entropy.

## 3. Interval Modeling

This section will present fundamental concepts associated with interval-valued calculus and interval-valued aggregation functions.

### 3.1. Interval-Valued Fuzzy Sets

Early in the 1970s, Zadeh developed IVFS to express ambiguity and uncertainty in practical applications [35]. They have since been applied in numerous areas, including decision making, recognizing patterns, and control systems [36,37,38,39].

The main distinguishing characteristic of IVFS is that each component of the universe of discourse is associated with an interval in which, according to epistemic interpretation, a given membership degree is placed, rather than a single membership degree [40]. This range reflects the degree of ambiguity or confusion surrounding the element’s degree of membership. As an example, the degree of proximity of a given temperature to being classified as “hot” or “cold” can be modeled using an IVFS. An interval, for example [0.6, 0.9], can be used to reflect the degree of uncertainty associated with the temperature’s membership degree rather than designating a single membership degree, such as “0.7”, to express the temperature’s proximity to being “hot”.

Interval Calculus is a mathematical framework designed for studying and analyzing real number intervals. Let $L^{I}$ be the set of all subintervals of the unit interval I=[0,1], where each interval can be represented by a pair of numbers $\left[\underline{x},\bar{x}\right]$, such that $\underline{x}\leq \bar{x}$. A partial classical order is a method for comparing two intervals based on their lower and upper bounds. The classical partial order in $L^{I}$ is defined as follows:

(1) $$\left[\underline{x},\bar{x}\right]⪯\left[\underline{y},\bar{y}\right]\Leftrightarrow \underline{x}\leq \underline{y},\bar{x}\leq \bar{y}$$

The join and meet operations are defined as:

(2) $$\left[\underline{x},\bar{x}\right]\vee \left[\underline{y},\bar{y}\right]=\left[\max\left(\underline{x},\underline{y}\right),\max\left(\bar{x},\bar{y}\right)\right],$$

(3) $$\left[\underline{x},\bar{x}\right]\wedge \left[\underline{y},\bar{y}\right]=\left[\min\left(\underline{x},\underline{y}\right),\min\left(\bar{x},\bar{y}\right)\right].$$

In terms of order $⪯,(L^{I},\vee ,\wedge )$ is a complete lattice (i.e., each subset of $L^{I}$ has supremum and infimum in $L^{I}$, cf. [35]). The values 0=[0,0] and 1=[1,1] represent the set’s lower and upper limits, respectively.

An interval-valued fuzzy set FinX is defined as a function $F:X\to L^{I},F\left(x\right)=\left[\underline{F}\left(x\right),\bar{F}\left(x\right)\right]\in L^{I}$ for x∈X, where $\underline{F}\left(x\right),\bar{F}\left(x\right)$ denote fuzzy sets. IVFS(X) denotes the collection of all intervals valued fuzzy sets in the universe *X*. In terms of the order ⪯, (IVFS(X),∨,∧) is a complete, bounded lattice.

Since the classically applied partial order between each pair of intervals is not linear (for example, [0.3,0.5] and [0.1,0.7] are incomparable with respect to the ⪯ order), diverse linear orders were also introduced for $L^{I}$ (cf. [21]). Well-known examples of linear orders are given below.

The Xu and Yager order $\left[\underline{x},\bar{x}\right]\leq_{XY}\left[\underline{y},\bar{y}\right]\;\mathrm{if}\;\mathrm{and}\;\mathrm{only}\;\mathrm{if}$:

(4) $$\underline{x}+\bar{x}<\underline{y}+\bar{y}\;\vee \;\left(\bar{x}+\underline{x}=\bar{y}+\underline{y},\;\bar{x}-\underline{x}⩽\bar{y}-\underline{y}\right).$$

The first lexicographical order $\left[\underline{x},\bar{x}\right]\leq_{\mathrm{Lex}1}\left[\underline{y},\bar{y}\right]\;\mathrm{if}\;\mathrm{and}\;\mathrm{only}\;\mathrm{if}$:

(5) $$\underline{x}<\underline{y}\;\vee \;\left(\underline{x}=\underline{y},\;\bar{x}\leq \bar{y}\right).$$

The second lexicographical order $\left[\underline{x},\bar{x}\right]\leq_{\mathrm{Lex}2}\left[\underline{y},\bar{y}\right]\;\mathrm{if}\;\mathrm{and}\;\mathrm{only}\;\mathrm{if}$:

(6) $$\bar{x}<\bar{y}\;\vee \;\left(\bar{x}=\bar{y},\;\underline{x}\leq \underline{y}\right).$$

An example comparison will be performed using the linear orders Lex1, Lex2, Xu and Yager for the intervals x=[0.2,0.4] and y=[0.1,0.5]. Since $\underline{x}+\bar{x}=0.2+0.4=0.6$, $\;\underline{y}+\bar{y}=0.1+0.5=0.6$, there is a need to check the second condition in order to compare the intervals with the Xu and Yager Order., i.e., the width of each interval need to be established. These widths are $x_{\omega }=0.4-0.2=0.2,\;y_{\omega }=0.5-0.1=0.4$. As a result, $x\leq_{XY}y$. Since $\underline{y}=0.1<\underline{x}=0.2$, $y\leq_{\mathrm{Lex}1}x$. Since $\bar{x}=0.4<\bar{y}=0.5$, $x\leq_{\mathrm{Lex}2}y$.

Diverse linear orders may yield diverse ordering of the intervals which are under consideration. However, some additional methods may be applied to decide about the order in the given applications (cf. [20]). The comparison of orders in Lex1 and Lex2 is based on the interval lower or upper bounds, while the comparison in the case of Xu and Yager order relays on considering both bounds. The condition $\underline{x}+\bar{x}=\underline{y}+\bar{y}$ is equivalent to $\frac{\underline{x}+\bar{x}}{2}=\frac{\underline{y}+\bar{y}}{2}$, namely the middle point representatives of each interval are compared, and if these values are equal, then in order to decide the order between intervals, the second option is to compare the width of the corresponding intervals. As a result, the Xu and Yager order seems to be more comprehensive in the analysis of the intervals to be compared. This is why, for the purpose of this contribution, this linear order was chosen to be applied in the algorithms studied. The Xu and Yager order proved to be more useful than Lex1 and Lex2 in some classification algorithms known from the literature (cf. [20], Chapter 6), which also justifies its choice.

### 3.2. Interval-Valued Aggregation Functions

Aggregation functions in [0,1] are applied to construct interval-valued aggregation functions in $L^{I}$. Aggregation function $A:{\left[0,1\right]}^{n}\to \left[0,1\right]$ (cf. [41]) is increasing with respect to each variable:

(7) $$\underset{1\leq i\leq n,x_{i},y_{i}\in \left[0,1\right]}{\forall }x_{i}\leq y_{i}\Rightarrow A\left(x_{1},\ldots ,x_{n}\right)\leq A\left(y_{1},\ldots ,y_{n}\right)$$

and satisfies the boundary conditions A(0,…,0)=0,A(1,…,1)=1.

An aggregating function *A* is called an averaging function if it satisfies the averaging property:

(8) $$\min\left(x_{1},\ldots ,x_{n}\right)\leq A\left(x_{1},\ldots ,x_{n}\right)\leq \max\left(x_{1},\ldots ,x_{n}\right),\;\;x_{1},\ldots ,x_{n}\in \left[0,1\right].$$

The most well-known aggregation function is the arithmetic mean:

(9) $$A\left(x_{1},\ldots ,x_{n}\right)=\frac{x_{1}+\ldots +x_{n}}{n},\;\;x_{1},\ldots ,x_{n}\in \left[0,1\right].$$

Various other aggregation functions are available, particularly the quasi-arithmetic means [41]. Analogously to the notion of an aggregation function in [0,1], the notion of an interval-valued aggregation function $A:{\left(L^{I}\right)}^{n}\to L^{I}$ is defined, i.e., A should fulfill the monotonicity condition (cf. (7)) and boundary conditions with the intervals 0=[0,0] and 1=[1,1]. However, due to the diversity of orders, interval-valued aggregation functions with respect to diverse orders may be defined (cf. [20]). The specifics of these relations are not the focus of the study, so the detailed information about the types of the orders is omitted here. The $\frac{0}{0}=0$ convention is used to calculate the $A_{3}$, $A_{4}$, $A_{8}$, and $A_{9}$ aggregations, which means that every division zero by zero within the calculations yields a value of zero. Let $x_{i}=\left[{\underline{x}}_{i},{\bar{x}}_{i}\right]$ for i=1,…,n. The list of applied in this research, interval-valued aggregation functions is the following:

(10) $$A_{1}\left(x_{1},x_{2},\ldots ,x_{n}\right)=\left[\frac{{\underline{x}}_{1}+{\underline{x}}_{2}+\ldots +{\underline{x}}_{n}}{n},\frac{{\bar{x}}_{1}+{\bar{x}}_{2}+\ldots +{\bar{x}}_{n}}{n}\right],$$

(11) $$A_{2}\left(x_{1},x_{2},\ldots ,x_{n}\right)=\left[\frac{{\underline{x}}_{1}+{\underline{x}}_{2}+\ldots +{\underline{x}}_{n}}{n},\max\left(\frac{{\underline{x}}_{1}+{\bar{x}}_{2}+\ldots +{\bar{x}}_{n}}{n},\ldots ,\frac{{\bar{x}}_{1}+\ldots +{\bar{x}}_{n-1}+{\underline{x}}_{n}}{n}\right)\right],$$

(12) $$A_{3}\left(x_{1},x_{2},\ldots ,x_{n}\right)=\left[\frac{{\underline{x}}_{1}+\ldots +{\underline{x}}_{n}}{n},\frac{{\bar{x}}_{1}^{2}+\ldots +{\bar{x}}_{n}^{2}}{{\bar{x}}_{1}+\ldots +{\bar{x}}_{n}}\right],$$

(13) $$A_{4}\left(x_{1},\ldots ,x_{n}\right)=\left[\frac{{\underline{x}}_{1}+\ldots +{\underline{x}}_{n}}{n},\frac{{\bar{x}}_{1}^{3}+\ldots +{\bar{x}}_{n}^{3}}{{\bar{x}}_{1}^{2}+\ldots +{\bar{x}}_{n}^{2}}\right].$$

(14) $$A_{5}\left(x_{1},\ldots ,x_{n}\right)=\left[\sqrt{\frac{{\underline{x}}_{1}^{2}+\ldots +{\underline{x}}_{n}^{2}}{n}},\sqrt[{3}]{\frac{{\bar{x}}_{1}^{3}+\ldots +{\bar{x}}_{n}^{3}}{n}}\right],$$

(15) $$A_{6}\left(x_{1},\ldots ,x_{n}\right)=\left[\sqrt[{3}]{\frac{{\underline{x}}_{1}^{3}+\ldots +{\underline{x}}_{n}^{3}}{n}},\sqrt[{4}]{\frac{{\bar{x}}_{1}^{4}+\ldots +{\bar{x}}_{n}^{4}}{n}}\right],$$

(16) $$A_{7}\left(x_{1},x_{2},\ldots ,x_{n}\right)=\left[\min\left(\frac{{\bar{x}}_{1}+{\underline{x}}_{2}+\ldots +{\underline{x}}_{n}}{n},\ldots ,\frac{{\underline{x}}_{1}+\ldots +{\underline{x}}_{n-1}+{\bar{x}}_{n}}{n}\right),\frac{{\bar{x}}_{1}+{\bar{x}}_{2}+\ldots +{\bar{x}}_{n}}{n}\right],$$

(17) $$A_{8}\left(x_{1},\ldots ,x_{n}\right)=\left[\sqrt[{n}]{{\underline{x}}_{1}\cdot \ldots \cdot {\underline{x}}_{n}},\frac{{\bar{x}}_{1}^{2}+\ldots +{\bar{x}}_{n}^{2}}{{\bar{x}}_{1}+\ldots +{\bar{x}}_{n}}\right],$$

(18) $$A_{9}\left(x_{1},\ldots ,x_{n}\right)=\left[\sqrt{\frac{{\underline{x}}_{1}^{2}+\ldots +{\underline{x}}_{n}^{2}}{n}},\frac{{\bar{x}}_{1}^{3}+\ldots +{\bar{x}}_{n}^{3}}{{\bar{x}}_{1}^{2}+\ldots +{\bar{x}}_{n}^{2}}\right],$$

(19) $$A_{10}\left(x_{1},\ldots ,x_{n}\right)=\left[\sqrt{\frac{{\underline{x}}_{1}^{2}+\ldots +{\underline{x}}_{n}^{2}}{n}},\sqrt{\frac{{\bar{x}}_{1}^{2}+\ldots +{\bar{x}}_{n}^{2}}{n}}\right].$$

In the proposed models, the aggregation functions that handle interval values are employed to amalgamate the intervals derived during the prediction phase. The application of these aggregations results in the formation of a consolidated interval, which offers a comprehensive depiction of the prediction.

To sum up, interval modeling is an effective tool for expressing ambiguity and uncertainty in practical applications. It is a useful tool for decision making, pattern recognition, as well as control systems because of the capability of handling imprecise and uncertain input more robustly than conventional fuzzy sets. Interval-valued membership degrees can be used to represent ambiguity and vagueness in a more flexible and nuanced manner, which can produce results that are more accurate and trustworthy [42].

## 4. Microarray Datasets

Microarray datasets are a type of data collection produced by microarray technology, a method that allows for the simultaneous measurement of expression levels of thousands of genes. Microarrays are small glass slides or chips that are covered with a grid of tiny dots, each representing a unique gene or DNA sequence.

These datasets often include genes and can contain tens of thousands of data points. They are commonly used in bioinformatics and genomics research to study patterns of gene expression and to identify potential biomarkers for diseases or other biological processes.

There are several unique characteristics of microarray datasets:

- They are typically high-dimensional and feature-rich, which can make them challenging to analyze and understand due to the difficulty in visualizing such large datasets and identifying patterns or trends.
- Gene expression levels are frequently correlated because genes involved in the same biological processes are often co-regulated. This can make it difficult to determine the direct contribution of each gene to a specific phenotype or disease.
- Microarray data can be noisy due to various sources of variability, including differences in sample preparation and labelling, the microarray technology itself, and errors in data collection and processing. This can make it challenging to identify strong patterns or trends in the data.

Despite these challenges, microarray datasets have been widely used in various research applications, such as studying gene expression, identifying biomarkers, and diagnosing diseases. They have also contributed to the development of personalized medicine approaches, where treatment plans are tailored to a patient’s unique genetic profile.

The following datasets (cf. [43]), described in Table 1, were applied in our experiments and are related to various medical specialities and research challenges. The identifiers for these datasets are provided in parentheses:

1. Identification of genetic subgroups in acute lymphoblastic leukaemia—Acute Lymphoblastic Leukaemia (ALL);
2. Human glioma—Brain Tumour (BTu);
3. Role of the chronic hepatitis C virus in the pathogenesis of HCV-associated hepatocellular carcinoma—Hepatitis C (HeC);
4. Transcription profiling of human heart samples with various causes of failure—Heart Failure Factors (HFF);
5. Study of genetic changes associated with skin psoriasis—Skin Psoriatic (SPs);
6. Profiling of critically ill children with sepsis, septic shock, and systemic inflammatory response syndrome (SIRS)—Septic Shock (SSh).

## 5. Details of Experiments and Methodology

The datasets applied in our experiments [43] were already described in the section devoted to microarray datasets.

The general method applied in the proposed three versions of the model is using an ensemble of heterogeneous classifiers. As was already mentioned in the Introduction, according to the literature, the selected classifiers (Random Forest, Support Vector Machines, or Multilayer Perceptrons) are believed to have good performance in diverse areas. Instead of optimizing the hyperparameters of each one, it seems to be a good approach to create an ensemble of them [14]. The implementation of the component classifiers was performed using scikit-learn library [44,45,46,47]. Moreover, a simple and well-known k-NN classifier was also involved as a component classifier to build the ensemble models. The hyperparameters of these models are the following. The first hyperparameter is interval order, which is default $\leq_{XY}$, other possible options are $\leq_{\mathrm{Lex}1}$ and $\leq_{\mathrm{Lex}2}$. The models’ hyperparameter refers to the selection of base models used in the ensemble. By default, this includes a variety of classifiers such as random forest with number of estimators set to {10, 50, 100}, multi-layer perceptron with hidden layer sizes set to {[100], [50, 50], [100, 50, 25]}, SVM with linear, polynomial, and radial basis function kernels, and k-NN with number of neighbors set to 1, 3, and 5, all using the Manhattan metric. The last hyperparameter is interval-valued aggregation, which can take on values from $A_{1}$ to $A_{10}$, with $A_{1}$ being the default, determines the method used to combine the predictions of the base models.

Further details of experiments are the following. The cross-validation (CV) strategy employed in this analysis utilizes a stratified train–test split [48] for cross-validation. The dataset is divided into training (80%) and testing (20%) sets. This split is repeated five times with different seeds to enhance the model’s robustness and provide a reliable performance estimate. In the case of the Proposed Entropy Model and Proposed Entropy Groups Model, the additional nested stratified two-fold cross-validation is applied to optimize the selection of the models using cross-entropy loss. The procedure is repeated 10 times with different random seeds, each time to obtain a series of results and test multiple distributions of train and test data. The nested cross-validation is executed on train data. It is worth noting that the hyperparameter optimization is only performed on train data and final evaluation of the model is performed on test data.

The microarrays dataset usually contains a low number of objects and additionally in the case of multi-class dataset the classes can be unbalanced and have little representatives. To keep the reasonable number of instances in nested cross-validation and to ensure that all classes will be present, the train dataset contains 80% of the data. The choice to use only two-fold in nested cross-validation is also based on these circumstances. The repetition of cross-validations with different seeds provides the representative results.

To scale the features, we use the min–max scaler [49]. This scaler transforms the features by scaling them to a given range, typically between 0 and 1.

### 5.1. The Role of Cross-Entropy Loss to Select Models

Cross-entropy is a measure of the difference between two probability distributions for a given random variable or set of events. It is built on the concept of entropy from information theory and it “is the average number of bits needed to encode data coming from a source with distribution p when we use model q” ([50], p. 57). The concept of logistic loss, or log loss, is related to cross-entropy. Even though these measures have different origins, they calculate the same quantity in the context of classification algorithms. To achieve good performance in a classification task, currently, a large number of learning algorithms rely on minimizing the cross-entropy loss ([51], p. 235) [52].

The log loss [53,54] is a measure of uncertainty or disorder, which is essentially what entropy measures. By minimizing the log loss, the model is effectively minimizing the entropy of the predictions, which means it is selecting the models that provide the most information (or the least uncertainty). This is where the connection between log loss and cross-entropy [52] becomes apparent: log loss is a form of cross-entropy. Cross-entropy is a measure of the difference between two probability distributions, and in this context, it is used to quantify the difference between the predicted and actual outcomes. Therefore, by minimizing log loss, we are effectively minimizing cross-entropy, leading to models that are more accurate and confident:

(20) $$CrossEntropyLoss_{\log}\left(Y,P\right)=-\log\mathrm{Pr}\left(Y|P\right)=-\frac{1}{N}\sum_{i=0}^{N-1}\sum_{k=0}^{K-1}y_{i,k}\log p_{i,k}$$

where [54]:

- *N* is the number of samples;
- *K* is the number of classes (labels);
- $P_{i,k}$ is the probability estimate for sample *i* belonging to class *k*;
- *Y* reflects the true labels encoded as a 1-of-K binary indicator matrix;
- $y_{i,k}$ is the binary indicator for whether sample *i* has label *k*.

The Proposed Entropy Model and Proposed Entropy Groups Model in the stage of fitting both use log loss to optimize the selection of models. Using the mentioned nested cross-validation, the strongest models, i.e., models with the lowest log loss, are recognized and kept in the models’ hyperparameters. The more details of this process are listed in Section 6.2 and Section 6.3.

### 5.2. Applied Metrics

Machine learning algorithms, known for their predictive and decision-making abilities, require evaluation metrics for effectiveness. Metrics such as accuracy [55], recall [56], precision [57], and F1 score [58] provide different perspectives on performance. Selecting the appropriate metric for a particular problem and evaluating a model’s performance can be aided by understanding these metrics. In our experiments, we used the following metrics: AUC [59], accuracy, recall, balanced accuracy [60], precision, and F1 score. Since we consider a multi-class problem, some of these metrics (recall, precision, and F1 score) are also available in specific types, such as micro, macro, and weighted.

We observed similar behavior between the results between the applied metrics, and this is why we only present the results for AUC, accuracy, and balanced accuracy (where accuracies were determined for the threshold value 0.5).

Accuracy [55] is a simple and intuitive metric used in machine learning to evaluate model performance, particularly in classification tasks. It is calculated by dividing the number of correct predictions by the total predictions. The following formula is used for calculating multi-class accuracy:

$$\begin{array}{cc} \mathrm{Multi}\text{-}\mathrm{class}\;\mathrm{Accuracy} & =\frac{\sum_{i=1}^{C}TP_{i}}{\sum_{i=1}^{C}\left(TP_{i}+FP_{i}+FN_{i}+TN_{i}\right)} \end{array}$$

where:

*C*—total number of classes;

*i*—each class’s index;

$TP_{i}$—true positives for class *i*;

$FP_{i}$—false positives for class *i*;

$FN_{i}$—false negatives for class *i*;

$TN_{i}$—true negatives for class *i*.

In multi-class classification, true positives are correct identifications of a class, false positives are incorrect attributions to a class, true negatives correctly identify non-membership, and false negatives incorrectly assume non-membership.

Balanced Accuracy [60] is a metric for evaluating classification models, particularly useful when classes are unbalanced. It provides a more detailed view of performance than traditional accuracy by considering the balance of classes. It is the average of recall scores for each class, assessing each class’s performance separately. The following formula is used for calculating multi-class Balanced Accuracy:

$$\begin{array}{cc} \mathrm{Multi}\text{-}\mathrm{class}\;\mathrm{Balanced}\;\mathrm{Accuracy} & =\frac{1}{C}\sum_{i=1}^{C}\frac{TP_{i}}{TP_{i}+FN_{i}}. \end{array}$$

The Receiver Operating Characteristic Area Under the Curve (ROC AUC) [59] is a performance measurement for classification problems at various threshold settings. ROC is a probability curve, and AUC is an area under ROC curve which tells how much a model is capable of distinguishing between classes.

For multi-class ROC AUC, either One-vs-Rest [61] (average ROC AUC for each class against all others) or One-vs-One [62] (average pairwise ROC AUC scores for each pair of classes) is used. The choice depends on the specific problem and number of classes. In this contribution, the One-vs-One is used.

## 6. Implementation of Proposed Models

In this section, three versions of the proposed models will be described. Each of them is based on interval modeling and two of them are using additionally the concept of cross-entropy. The implementations of the proposed models are available at the repository [63].

### 6.1. Fitting Process of the Proposed Model

The input of the ensemble model is a collection $ModelGroups=[MG_{1},\ldots ,MG_{n}],n\geq 2$, where each model group $MG_{i}=\left[M_{1},\ldots ,M_{m}\right],i\in \left\{1,\ldots ,n\right\},m\geq 2$ consist of a collection of independent models. The procedure for fitting the models [64] encompasses several stages

1. For each model group MG in ModelGroups:
  (a) For each model *M* in model group MG:
    - Fit model *M* on train data.

The ensemble model is a collection of independent models, grouped into distinct sets. Each model is trained individually on the data. The trained models, organized in their respective groups, form the ensemble model, which is used for constructing intervals.

### 6.2. Fitting Process of the Proposed Entropy Model

The input of the ensemble model is a collection $ModelGroups=[MG_{1},\ldots ,MG_{n}],n\geq 2$, where each model group $MG_{i}=\left[M_{1},\ldots ,M_{m}\right],i\in \left\{1,\ldots ,n\right\},m\geq 2$ consist of a collection of independent models. The procedure for fitting the models [65] encompasses several stages:

1. For each model group MG in ModelGroups:
  (a) Find the optimal subset of MG by performing the following procedure:
  (b) Repeat 10-times-stratified two-fold cross-validation, split train data TRD into cross-validation train data $TRD_{CV}$ and cross-validation test data $TSD_{CV}$:
    - For each model *M* in model group MG:
      - Fit model *M* on cross-validation train data $TRD_{CV}$;
      - Calculate the cross-entropy loss on cross-validation test data $TSD_{CV}$.
  (c) Compute the mean cross-entropy loss for each model *M* in MG based on all folds in two-fold cross-validation;
  (d) Compute the mean cross-entropy loss of a group MG based on the means computed in the previous step;
  (e) Select the top two models with the lowest mean cross-entropy loss in group MG;
  (f) Append additional models with mean cross-entropy loss less than or equal to the average cross-entropy loss of the group MG, if they exist.

The enhanced ensemble model is composed of independent models, divided into groups. Each group undergoes an optimization process where the top-performing models are selected based on their mean cross-entropy loss from stratified two-fold cross-validation on training data. The final ensemble model is a compilation of these optimized groups. The cross-entropy loss serves as a key metric in this process, guiding the selection of models within each group to ensure the best performance.

### 6.3. Fitting Process of the Proposed Entropy Groups Model

The input of the ensemble model is a collection $ModelGroups=[MG_{1},\ldots ,MG_{n}],n\geq 2$, where each model group $MG_{i}=\left[M_{1},\ldots ,M_{m}\right],$i∈{1,…,n},m≥2 consist of a collection of independent models. The procedure for fitting the models [66] encompasses several stages:

1. For each model group MG in ModelGroups:
  (a) Find the optimal subset of MG by performing the following procedure:
  (b) Repeat 10-times-stratified two-fold cross-validation, split train data TRD into cross-validation train data $TRD_{CV}$ and cross-validation test data $TSD_{CV}$:
    - For each model *M* in model group MG:
      - Fit model *M* on cross-validation train data $TRD_{CV}$;
      - Calculate the cross-entropy loss on cross-validation test data $TSD_{CV}$.
  (c) Compute the mean cross-entropy loss for each model *M* in MG based on all folds in two-fold cross-validation;
  (d) Compute the mean cross-entropy loss of a group MG based on the means computed in the previous step;
  (e) Select the top two models with the lowest mean cross-entropy loss in group MG;
  (f) Append additional models with mean cross-entropy loss less than or equal to the average cross-entropy loss of the group MG, if they exist;
  (g) Compute the mean cross-entropy loss of all groups ModelGroups based on the means computed in step (d);
  (h) Select the top two groups with the lowest mean cross-entropy loss;
  (i) Append additional groups with mean cross-entropy loss less than or equal to the average cross-entropy loss of all groups ModelGroups, if they exist.

As a result, the ensemble model consists of fitted independent models, divided into arbitrary groups. The cross-entropy loss is used here to optimize the selection of independent models in each group. Additionally, if some whole group is not optimal, i.e., it is lowering the overall performance of a classifier, it will be removed.

### 6.4. Process of Predicting Decision Classes Using the Proposed Models

The input of the ensemble model is a collection $ModelGroups=[MG_{1},\ldots ,MG_{n}],n\geq 2$, where each model group $MG_{i}=\left[M_{1},\ldots ,M_{m}\right],i\in \left\{1,\ldots ,n\right\},m\geq 2$ consist of a collection of independent models; the interval-valued aggregation A, the interval order *o*. The prediction procedure is carried out through the following stages:

1. Create empty collection of intervals ivs;
2. For each model group MG in ModelGroups:
  (a) Create empty collection of soft labels $sls_{c}$ for each decision class *c*;
  (b) For each model *M* in model group MG:
    - Compute model *M* soft labels sl for each decision class *c* on test data and append it to $sls_{c}$;
    - If soft labels values are outside unit interval [0,1], then normalize it using the softmax function;
  (c) For each decision class *c*, create intervals $i_{c}=\left[\min sls_{c},\max sls_{c}\right]$ and append it to ivs.
3. For each class *c* aggregate its corresponding intervals $i_{c}$ from ivs using A;
4. Sort the intervals using order *o*;
5. Return the class *c*, the corresponding interval of which is the highest in the term of order *o*.

Soft labels indicate the degree of membership of the data to the given classes, while hard labels indicate the belonging only to one, concrete class. Soft labels represent the extent to which data associate with various classes, whereas hard labels denote the data’s affiliation with a single, specific class.

### 6.5. Process of Predicting Decision Class Membership of Proposed Models

The input of the ensemble model is a collection $ModelGroups=[MG_{1},\ldots ,MG_{n}],n\geq 2$, where each model group $MG_{i}=\left[M_{1},\ldots ,M_{m}\right],i\in \left\{1,\ldots ,n\right\},m\geq 2$ consist of a collection of independent models, the interval-valued aggregation A, and the interval order *o*. The prediction procedure is carried out through the following stages:

1. Create empty collection of intervals ivs;
2. For each model group MG in ModelGroups:
  (a) Create empty collection of soft labels $sls_{c}$ for each decision class *c*;
  (b) For each model *M* in model group MG:
    - Compute model *M* soft labels sl for each decision class *c* on test data and append it to $sls_{c}$;
    - If soft labels values are outside unit interval [0,1], then normalize it using the softmax function.
  (c) For each decision class *c*, create intervals $i_{c}=\left[\min sls_{c},\max sls_{c}\right]$ and append it to ivs.
3. For each class *c*, aggregate its corresponding intervals $i_{c}$ from ivs using A;
4. For each class *c*, compute its membership degree by averaging the lower and upper bound of an interval assigned to *c*;
5. If membership degrees of all decision classes are not sum to value 1, then normalize them;
6. Return the membership degrees for all decision classes.

In Table 2, the comparison of the considered models is presented.

## 7. Results

In the following tables, we use several abbreviations to refer to different machine learning models and methods. Here is a brief introduction to these abbreviations:

- POM_eGRP—Proposed Model Entropy Groups;
- POM_ENT—Proposed Model Entropy;
- POM_ALG—Proposed Model;
- SVC—Support Vector Classification;
- RND_FST—Random Forest;
- MLP—Multilayer Perceptron;
- BAGGING—Bagging;
- 7NN_MUL—Seven Nearest Neighbors;
- 7NN_OVR—Seven Nearest Neighbors; (One-vs-Rest);
- 7NN_OVO—Seven Nearest Neighbors (One-vs-One);
- 5NN_MUL—Five Nearest Neighbors
- 5NN_OVR—Five Nearest Neighbors (One-vs-Rest);
- 5NN_OVO—Five Nearest Neighbors (One-vs-One);
- 3NN_MUL—Three Nearest Neighbors;
- 3NN_OVR—Three Nearest Neighbors (One-vs-Rest);
- 3NN_OVO—Three Nearest Neighbors (One-vs-One);
- 1NN_MUL—One Nearest Neighbor;
- 1NN_OVR—One Nearest Neighbor (One-vs-Rest);
- 1NN_OVO—One Nearest Neighbor (One-vs-One).

The results are presented in descending order with respect to the ROC AUC measure. We decided to analyze the results with respect to this measure, since it is related to diverse thresholds, unlike accuracy or balanced accuracy, which is determined with respect to a given threshold. It is natural that results for accuracy may have lower values comparing to AUC ROC—the considered threshold (here 0.5) may not be optimal. On the other hand, balanced accuracy results may have lower values than those of accuracy due to the imbalance problem which occur in the considered datasets.

Some of the results in Table 3, Table 4, Table 5, Table 6, Table 7 and Table 8 are highlighted. Light gray color denotes the highest three results of the models which are used as comparative models, i.e., these are all component models used to create an ensemble model (Random Forest, MLP, SVM, and k-NN with diverse values of k); additionally. this is a well-known ensemble model, which is Bagging and OVO and OVA versions of suitable k-NN models. Light green, light blue, and light red colors denote the highest three results of the proposed models, with corresponding versions. The results for the same aggregation function, applied as a hyperparameter of the model, are highlighted in the same color.

The results for the proposed models will be analyzed for a given hyperparameter value, which is an aggregation function, as is highlighted in light green, light blue, and light red colors in Table 3, Table 4, Table 5, Table 6, Table 7 and Table 8.

For the dataset ALL, Random Forest was the best-performing classifier. The Proposed Model Entropy Groups has very similar results to Random Forest, but it is several percentage points better than the Proposed Model Entropy, and in turn, it is also better than the Proposed Model (without the concept of cross-entropy).

For the dataset Btu, all versions of the considered models obtained better results than the component models or the models applied as comparative ones. However, the difference between the best result which belongs to the Proposed Model Entropy Groups (with the aggregation $A_{7}$) and ex aequo for the Proposed Model (with the aggregation $A_{4}$) is only slightly better than the best results for other models, i.e., Random Forest.

For the dataset HeC, there are only slight differences (about one percentage point between the best result of the Proposed Model and the best results of the comparative models) between the considered three models and the best comparative models, i.e., Random Forest and Bagging. This time, the Proposed Model gave slightly better results than the proposed models with the applied cross-entropy.

For the dataset HFF, The Proposed Model Entropy Groups has the best results—seven percentage points better than Random Forest (the best comparative model), about three percentage points better than Proposed Model Entropy; moreover, it is also better than Proposed Model (without the concept of cross-entropy). In the case of this dataset, the differences between the results of the proposed models and other comparative models are the most significant.

For the dataset SPs, there are only slight differences (third decimal place between the best result of the proposed models and the best results of the comparative model Random Forest and Bagging and also between the proposed models). However, the best performance was obtained for the Bagging model and the proposed models with cross-entropy had better performance than the Proposed Model (without cross-entropy).

For the dataset SSh, the Proposed Model Entropy has the best results—five percentage points better than Support Vector Machine (the best comparative model) and about two percentage points better than Proposed Model. In the case of this dataset, the differences between the results of the proposed models and other comparative models are significant. Moreover, among the three proposed models the Proposed Model Entropy has the best results, the Proposed Model Entropy Groups has the worst results. All results of the proposed models are better than those of the comparative models.

## 8. Discussion

Regarding the interval-valued aggregation function which was applied here as a hyperparameter, we may notice that the choice of the aggregation to obtain the best performance of the classifier depends on the dataset (diverse aggregation functions yield the best results in each dataset). It confirms that it is right to include diverse aggregations in the proposed models.

Regarding the choice of the model, for most of the datasets, the best results were obtained by the Proposed Model Entropy Groups, but there are also datasets for which the Proposed Model Entropy gave the best results. However, the cross-entropy applied to choose the best component classifiers seems undoubtedly a good choice to improve the classification results.

**Figure 1:**
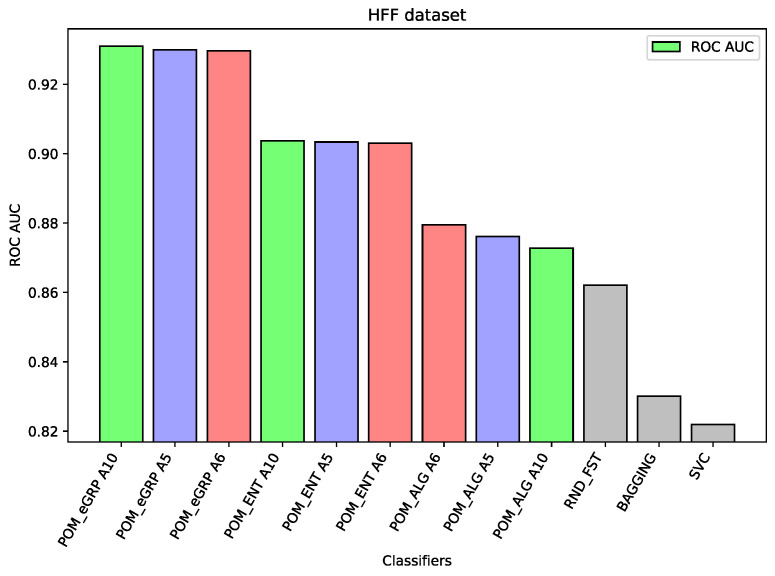
Performance of the selected models for the HFF dataset.

**Figure 2:**
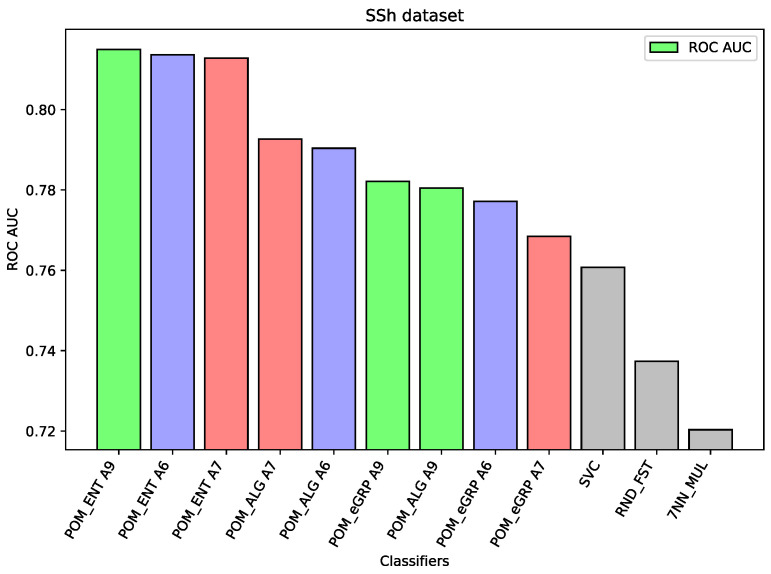
Performance of the selected models for the SSh dataset.

Analyzing the results on each dataset, we may notice that if the component classifiers such as Random Forest, MLP, or SVC have relatively weak results, as in the case of the dataset HFF (cf. Figure 1) or the dataset SSh (cf. Figure 2), then all the proposed ensemble models obtain much better results than the component models or other comparative models. In the case of the HFF dataset, the Proposed Model Entropy Group obtained the best result 0.931 ROC AUC, while the highest result for the component models belong to Random Forest, i.e., 0.862 ROC AUC. Moreover, in the case of this dataset, we may observe a clear dependence between the three considered models—the best results (regardless of the interval-valued aggregation function applied) were obtained by the Proposed Model Entropy Group, then by Proposed Model Entropy, and finally by the Proposed Model. In the case of the SSh dataset, the Proposed Model Entropy obtained the highest result, which is 0.815 ROC AUC, while the highest result for the component models belong to SVC, i.e., 0.761 ROC AUC. Moreover, in the case of this dataset, we may observe a clear dominant model among the three considered models—the best results (regardless the interval-valued aggregation function applied) were obtained by the Proposed Model Entropy.

**Figure 3:**
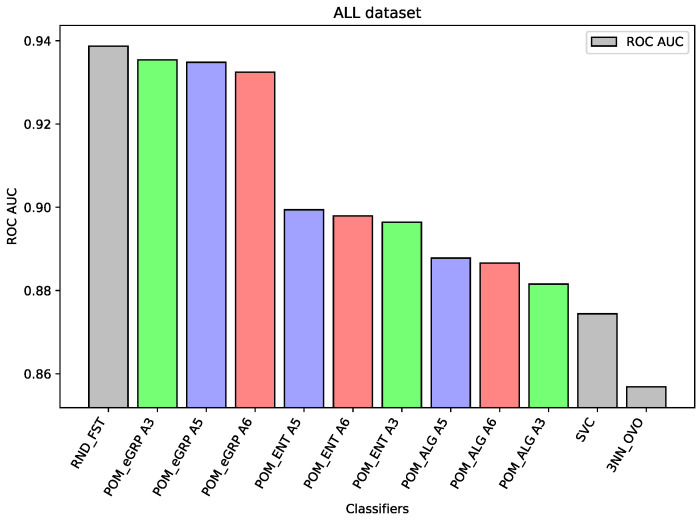
Performance of the selected models for the ALL dataset.

**Figure 4:**
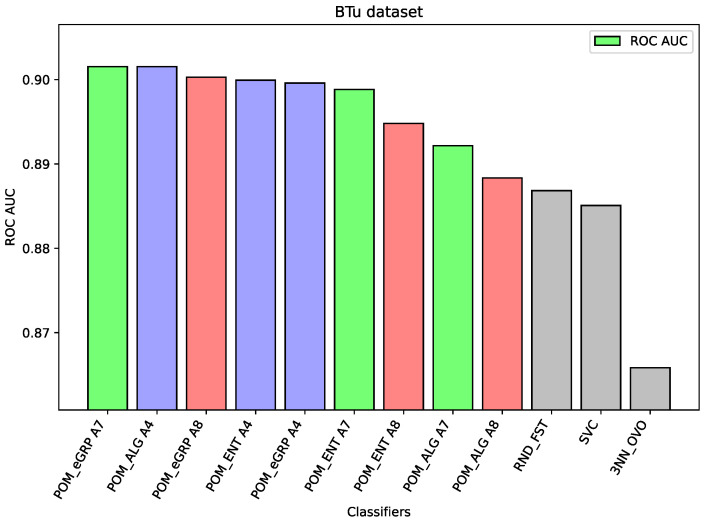
Performance of the selected models for the BTu dataset.

**Figure 5:**
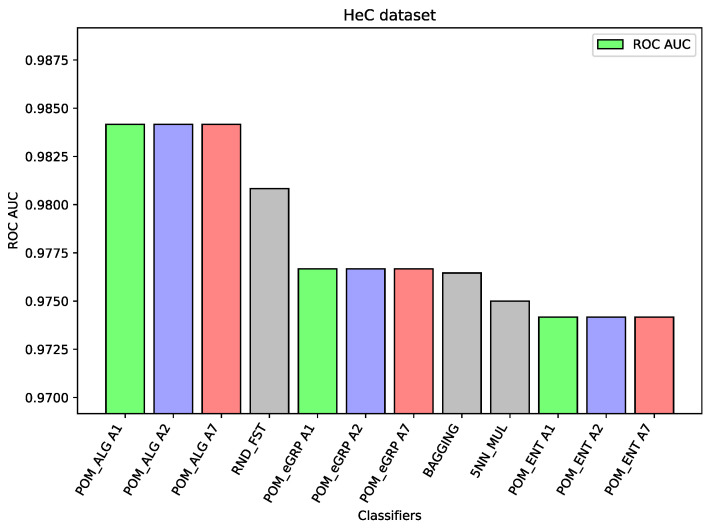
Performance of the selected models for the HeC dataset.

**Figure 6:**
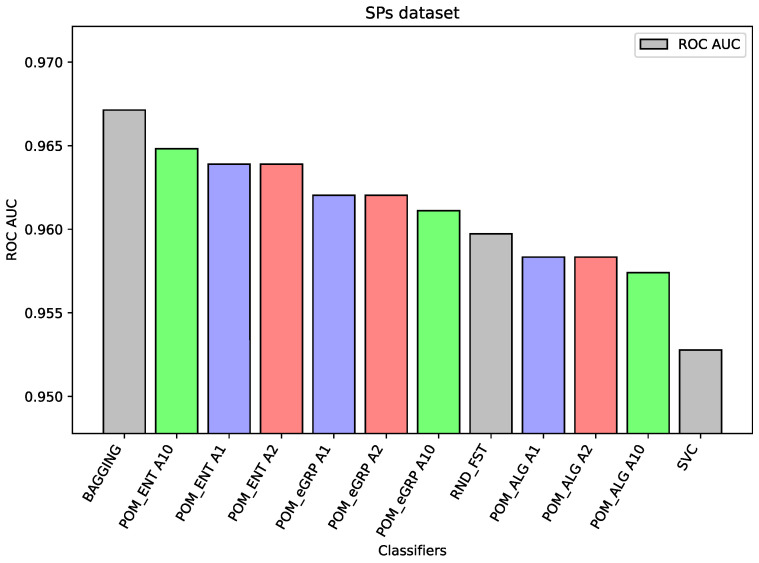
Performance of the selected models for the SPs dataset.

In the case of the ALL dataset (cf. Figure 3), the component classifier which is Random Forest was strong enough to obtain an ROC AUC value of 0.939. As a result, ensemble models were weaker, and it proves the well-known thesis that creating ensembles makes sense in the case of the weak performance of single-component classifiers. A similar situation is in the case of the BTu dataset (cf. Figure 4). On this dataset, the proposed ensemble models had better performance than the component models, but the difference was small. The ROC AUC result for Random Forest is 0.887 or 0.885 for SVC, which may be considered a high value, so the ensemble models were only able to improve these results slightly. Again, analogous behavior may be observed for the dataset HeC (cf. Figure 5), where the best component model ROC AUC result belongs to Random Forest 0.981 and the Proposed Model was only able to improve it to the value 0.984. Similarly, on the dataset SPs (cf. Figure 6), the differences between the proposed models and the best comparative models are very small—for most of the cases, this is the difference in the third decimal place. The best value of the proposed model is 0.965 ROC AUC and the best value of the component model belongs to Random Forest, i.e., 0.960 ROC AUC (which is a very high value).

The following Figure 1, Figure 2, Figure 3, Figure 4, Figure 5 and Figure 6 present the performance of the selected models—the best three results of each proposed model and the best three results of the comparative models (as highlighted in light green, light blue, light red, and light gray colors in Table 3, Table 4, Table 5, Table 6, Table 7 and Table 8).

Following the results of the non-parametric Kruskal–Wallis test [67] using a 0.05 significance level and Dunn’s Multiple Comparison test with Bonferroni correction [68], we can further discuss the performance of the models (with the use of AUC measure). In Table 9, Table 10 and Table 11, the results of the Kruskal–Wallis test and Dunn’s Multiple Comparison test with Bonferroni correction [68] are presented. Each of compared groups consist of 10 values AUC, obtained for 10 interval-valued aggregation functions and each model. The results indicate that there is a statistically significant difference in the AUC values across the three proposed models POM_ALG, POM_ENT, and POM_eGRP. This is evident as the *p*-value of the Kruskal–Wallis test is less than 0.05 for each dataset, suggesting that there exists at least one pair of models which has a significant difference in results. Upon applying Dunn’s test with Bonferroni corrections as a post hoc test, it was observed that for most of the considered datasets, the performance of a model with the cross-entropy involved (POM_ENT or POM_eGRP) is significantly better than the performance of the model POM_ALG. The exception is the HeC dataset where POM_ALG obtained the best results. In Table 9, Table 10 and Table 11 the *p*-values, i.e., the significant differences, are in bold (concrete values of AUC are presented in Table 3, Table 4, Table 5, Table 6, Table 7 and Table 8). The most clear situation is in the case of the HFF dataset, where there are significant difference between each pair of the considered models POM_ALG, POM_ENT, and POM_eGRP. According to Table 6 (where the AUC results for the HFF dataset are provided), the best performing model is POM_eGRP, then POM_ENT, and finally POM_ALG. The obtained results indicate that the incorporation of cross-entropy in the models in most of the cases enhances their performance. However, there is no clear tendency to suggest which of the models POM_ENT or POM_eGRP could be considered a better solution. The performance varies across different datasets. In some datasets, POM_eGRP outperforms POM_ENT, while in others, the reverse is true. This suggests that the choice between POM_ENT and POM_eGRP may depend on the specific characteristics of the dataset. Further research could be conducted to investigate the factors that influence the performance of POM_ENT and POM_eGRP on different datasets, which could provide insights into the conditions under which each model is most effective. This could potentially lead to the development of more robust and versatile models for interval-valued classification.

## 9. Conclusions

To sum up, in this research, the problem of using interval modeling and cross-entropy in ensemble heterogeneous classification was applied. Interval modeling proved to be a useful tool in improving classification results for high-dimensional datasets such as microarrays compared to the results of component classifiers, Multilayer Perceptron, Support Vector Classification, and Random Forest, which usually provide good performance for diverse problems. Applying cross-entropy to choose the best component classifier for the ensemble was even able to improve these results. Two models with the cross-entropy involved were analyzed. In the case of most of the considered datasets, the differences between the proposed model (using interval modeling) and at least one of the models with the cross-entropy (applied to choose the best components in the ensemble) are statistically significant.

In future work, other approaches of using interval modeling and applying the concept of cross-entropy is planned for other problems of classification, such as numerous missing values in datasets or coping with imbalanced datasets. Moreover, for the presented models, it is important to adjust the considered ensemble models to the case of other types of entropy, for example Tsallis, Renyi, Shannon, Kolmogorov-Sinai, or approximate entropy (cf. [69,70,71,72,73,74]). The applied entropy measure may have an influence on the overall results of the considered models.

## Figures and Tables

**Table 1:** Characteristics of the datasets.

Dataset	Samples	Genes	Classes (and Class Distribution)
ALL	190	22,277	7 (0.23, 0.23, 0.23, 0.19, 0.07, 0.03, 0.02)
BTu	180	54,613	4 (0.45, 0.28, 0.14, 0.13)
HeC	124	22,277	4 (0.38, 0.33, 0.15, 0.14)
HFF	210	22,283	3 (0.51, 0.41, 0.08)
SPs	180	54,675	3 (0.36, 0.32, 0.32)
SSh	227	54,675	5 (0.47, 0.23, 0.12, 0.11, 0.07)

**Table 2:** Comparison of the considered models.

Model	Characteristics
Proposed Model	No optimization of the independent model selection.
Proposed Entropy Model	Employs a repeated two-fold cross-validation to select the optimal models within each group
Proposed Entropy Groups Model	Employs a repeated two-fold cross-validation for optimal model selection within groups. Applies the same cross-validation process for superior selection of model groups.

**Table 3:** Performance measures of the considered models for the ALL dataset.

Classifier	Aggregation	ROC AUC	Accuracy	Balanced Accuracy
RND_FST		0.939±0.050	0.779±0.021	0.529±0.014
POM_eGRP	$A_{3}$	0.935 ± 0.051	0.811±0.054	0.614±0.083
POM_eGRP	$A_{5}$	0.935±0.051	0.811±0.054	0.614±0.083
POM_eGRP	$A_{6}$	0.932±0.053	0.811±0.054	0.614±0.083
POM_eGRP	$A_{8}$	0.932±0.049	0.726±0.131	0.536±0.126
POM_eGRP	$A_{1}$	0.932±0.047	0.800±0.039	0.586±0.066
POM_eGRP	$A_{7}$	0.932±0.047	0.800±0.039	0.586±0.066
POM_eGRP	$A_{10}$	0.930±0.046	0.811±0.054	0.614±0.083
POM_eGRP	$A_{2}$	0.930±0.046	0.800±0.039	0.586±0.066
POM_eGRP	$A_{9}$	0.930±0.057	0.811±0.054	0.614±0.083
POM_eGRP	$A_{4}$	0.928±0.059	0.779±0.052	0.571±0.078
POM_ENT	$A_{5}$	0.899±0.042	0.789±0.067	0.600±0.089
POM_ENT	$A_{6}$	0.898±0.044	0.789±0.067	0.600±0.089
POM_ENT	$A_{10}$	0.898±0.035	0.800±0.061	0.629±0.074
POM_ENT	$A_{3}$	0.896 ± 0.048	0.789±0.067	0.600±0.089
POM_ENT	$A_{1}$	0.894±0.040	0.800±0.061	0.629±0.074
POM_ENT	$A_{2}$	0.894±0.040	0.800±0.061	0.629±0.074
POM_ENT	$A_{7}$	0.894±0.040	0.800±0.061	0.629±0.074
POM_ENT	$A_{4}$	0.893±0.053	0.779±0.077	0.593±0.097
POM_ALG	$A_{1}$	0.892±0.051	0.811±0.054	0.636±0.073
POM_ALG	$A_{2}$	0.892±0.051	0.811±0.054	0.636±0.073
POM_ALG	$A_{7}$	0.892±0.051	0.811±0.054	0.636±0.073
POM_ENT	$A_{9}$	0.892±0.052	0.789±0.067	0.600±0.089
POM_ALG	$A_{10}$	0.890±0.057	0.811±0.054	0.636±0.073
POM_ALG	$A_{5}$	0.888±0.059	0.811±0.054	0.636±0.073
POM_ALG	$A_{6}$	0.887±0.059	0.800±0.052	0.629±0.070
POM_ALG	$A_{3}$	0.882 ± 0.064	0.768±0.054	0.650±0.061
POM_ALG	$A_{9}$	0.880±0.066	0.747±0.039	0.636±0.061
POM_ENT	$A_{8}$	0.877±0.042	0.747±0.112	0.571±0.117
POM_ALG	$A_{4}$	0.875±0.061	0.747±0.052	0.657±0.080
SVC		0.874±0.053	0.768±0.054	0.521±0.036
POM_ALG	$A_{8}$	0.865±0.065	0.747±0.052	0.636±0.102
3NN_OVO		0.857±0.065	0.705±0.042	0.564±0.069
BAGGING		0.840±0.077	0.811±0.063	0.657±0.105
7NN_MUL		0.831±0.045	0.674±0.039	0.521±0.066
7NN_OVR		0.830±0.044	0.684±0.047	0.507±0.061
3NN_OVR		0.826±0.038	0.737±0.067	0.564±0.089
3NN_MUL		0.826±0.038	0.684±0.033	0.550±0.048
5NN_OVR		0.824±0.051	0.716±0.054	0.550±0.066
5NN_MUL		0.824±0.051	0.695±0.039	0.557±0.066
1NN_MUL		0.800±0.047	0.747±0.052	0.657±0.080
1NN_OVR		0.800±0.047	0.747±0.052	0.657±0.080
1NN_OVO		0.799±0.042	0.747±0.052	0.657±0.080
MLP		0.795±0.038	0.242±0.063	0.164±0.043
7NN_OVO		0.792±0.048	0.684±0.047	0.507±0.073
5NN_OVO		0.791±0.041	0.705±0.054	0.543±0.083

**Table 4:** Performance measures of the considered models for the BTu dataset.

Classifier	Aggregation	ROC AUC	Accuracy	Balanced Accuracy
POM_eGRP	$A_{7}$	0.902±0.029	0.733±0.054	0.705±0.070
POM_ALG	$A_{4}$	0.902±0.029	0.722±0.050	0.695±0.071
POM_eGRP	$A_{8}$	0.900±0.023	0.722±0.061	0.688±0.078
POM_ENT	$A_{4}$	0.900±0.027	0.733±0.065	0.713±0.075
POM_eGRP	$A_{4}$	0.900±0.021	0.722±0.061	0.688±0.078
POM_eGRP	$A_{1}$	0.899±0.027	0.733±0.054	0.705±0.070
POM_eGRP	$A_{2}$	0.899±0.027	0.733±0.054	0.705±0.070
POM_ENT	$A_{9}$	0.899±0.028	0.733±0.065	0.713±0.075
POM_ENT	$A_{7}$	0.899±0.037	0.756±0.057	0.746±0.051
POM_eGRP	$A_{10}$	0.899±0.024	0.733±0.054	0.705±0.070
POM_eGRP	$A_{3}$	0.898±0.024	0.722±0.061	0.688±0.078
POM_eGRP	$A_{9}$	0.898±0.020	0.722±0.061	0.688±0.078
POM_ALG	$A_{3}$	0.898±0.031	0.744±0.075	0.730±0.075
POM_ALG	$A_{9}$	0.898±0.031	0.733±0.082	0.720±0.081
POM_eGRP	$A_{6}$	0.898±0.021	0.722±0.061	0.688±0.078
POM_ENT	$A_{1}$	0.898±0.036	0.756±0.057	0.746±0.051
POM_ENT	$A_{2}$	0.898±0.036	0.756±0.057	0.746±0.051
POM_eGRP	$A_{5}$	0.898±0.024	0.722±0.061	0.688±0.078
POM_ENT	$A_{3}$	0.897±0.032	0.744±0.057	0.730±0.060
POM_ENT	$A_{6}$	0.897±0.034	0.744±0.057	0.730±0.060
POM_ENT	$A_{5}$	0.897±0.033	0.744±0.057	0.730±0.060
POM_ALG	$A_{10}$	0.896±0.022	0.744±0.075	0.730±0.075
POM_ENT	$A_{8}$	0.895±0.032	0.722±0.061	0.688±0.078
POM_ENT	$A_{10}$	0.894±0.032	0.744±0.057	0.730±0.060
POM_ALG	$A_{5}$	0.894±0.025	0.744±0.075	0.730±0.075
POM_ALG	$A_{6}$	0.894±0.026	0.744±0.075	0.730±0.075
POM_ALG	$A_{1}$	0.893±0.023	0.744±0.075	0.730±0.075
POM_ALG	$A_{2}$	0.892±0.023	0.744±0.075	0.730±0.075
POM_ALG	$A_{7}$	0.892±0.022	0.744±0.075	0.730±0.075
POM_ALG	$A_{8}$	0.888±0.036	0.711±0.042	0.692±0.065
RND_FST		0.887±0.020	0.700±0.075	0.665±0.072
SVC		0.885±0.036	0.689±0.057	0.650±0.040
3NN_OVO		0.866±0.019	0.667±0.099	0.672±0.094
7NN_OVR		0.857±0.033	0.667±0.079	0.644±0.077
7NN_MUL		0.856±0.034	0.700±0.075	0.701±0.076
5NN_OVO		0.852±0.010	0.700±0.075	0.701±0.076
BAGGING		0.846±0.043	0.700±0.075	0.672±0.082
5NN_OVR		0.840±0.041	0.700±0.075	0.694±0.076
5NN_MUL		0.840±0.041	0.678±0.074	0.695±0.068
3NN_MUL		0.838±0.035	0.689±0.090	0.705±0.079
3NN_OVR		0.838±0.035	0.667±0.099	0.672±0.094
7NN_OVO		0.826±0.033	0.678±0.096	0.647±0.092
1NN_OVO		0.814±0.025	0.622±0.082	0.638±0.101
MLP		0.783±0.128	0.467±0.242	0.523±0.199
1NN_MUL		0.758±0.067	0.622±0.082	0.638±0.101
1NN_OVR		0.758±0.067	0.622±0.082	0.638±0.101

**Table 5:** Performance measures of the considered models for the HeC dataset.

Classifier	Aggregation	ROC AUC	Accuracy	Balanced Accuracy
POM_ALG	$A_{1}$	0.984±0.027	0.954±0.062	0.938±0.079
POM_ALG	$A_{2}$	0.984±0.027	0.954±0.062	0.938±0.079
POM_ALG	$A_{7}$	0.984±0.027	0.954±0.062	0.938±0.079
RND_FST		0.981±0.021	0.892±0.092	0.850±0.129
POM_ALG	$A_{3}$	0.977±0.042	0.954±0.062	0.938±0.079
POM_ALG	$A_{4}$	0.977±0.042	0.954±0.062	0.938±0.079
POM_ALG	$A_{5}$	0.977±0.042	0.954±0.062	0.938±0.079
POM_ALG	$A_{6}$	0.977±0.042	0.954±0.062	0.938±0.079
POM_ALG	$A_{9}$	0.977±0.042	0.954±0.062	0.938±0.079
POM_ALG	$A_{10}$	0.977±0.042	0.954±0.062	0.938±0.079
POM_ALG	$A_{8}$	0.977±0.042	0.938±0.058	0.925±0.073
POM_eGRP	$A_{1}$	0.977±0.034	0.969±0.038	0.950±0.061
POM_eGRP	$A_{2}$	0.977±0.034	0.969±0.038	0.950±0.061
POM_eGRP	$A_{7}$	0.977±0.034	0.969±0.038	0.950±0.061
BAGGING		0.976±0.019	0.846±0.084	0.792±0.069
POM_ENT	$A_{3}$	0.976±0.048	0.969±0.038	0.950±0.061
POM_ENT	$A_{4}$	0.976±0.048	0.969±0.038	0.950±0.061
POM_ENT	$A_{5}$	0.976±0.048	0.969±0.038	0.950±0.061
POM_ENT	$A_{6}$	0.976±0.048	0.969±0.038	0.950±0.061
POM_ENT	$A_{8}$	0.976±0.048	0.969±0.038	0.950±0.061
POM_ENT	$A_{9}$	0.976±0.048	0.969±0.038	0.950±0.061
5NN_MUL		0.975±0.047	0.954±0.038	0.938±0.056
5NN_OVR		0.975±0.047	0.938±0.058	0.943±0.069
POM_eGRP	$A_{3}$	0.975±0.035	0.969±0.038	0.950±0.061
POM_eGRP	$A_{4}$	0.975±0.035	0.969±0.038	0.950±0.061
POM_eGRP	$A_{5}$	0.975±0.035	0.969±0.038	0.950±0.061
POM_eGRP	$A_{6}$	0.975±0.035	0.969±0.038	0.950±0.061
POM_eGRP	$A_{8}$	0.975±0.035	0.969±0.038	0.950±0.061
POM_eGRP	$A_{9}$	0.975±0.035	0.969±0.038	0.950±0.061
POM_eGRP	$A_{10}$	0.975±0.035	0.969±0.038	0.950±0.061
3NN_MUL		0.975±0.045	0.938±0.075	0.938±0.079
3NN_OVR		0.975±0.045	0.908±0.090	0.918±0.083
POM_ENT	$A_{1}$	0.974±0.047	0.969±0.038	0.950±0.061
POM_ENT	$A_{2}$	0.974±0.047	0.969±0.038	0.950±0.061
POM_ENT	$A_{7}$	0.974±0.047	0.969±0.038	0.950±0.061
POM_ENT	$A_{10}$	0.974±0.047	0.969±0.038	0.950±0.061
1NN_OVO		0.974±0.019	0.923±0.049	0.925±0.047
3NN_OVO		0.969±0.060	0.938±0.075	0.938±0.079
7NN_OVR		0.969±0.053	0.938±0.058	0.943±0.069
7NN_MUL		0.969±0.053	0.923±0.049	0.915±0.065
SVC		0.967±0.047	0.908±0.058	0.875±0.068
5NN_OVO		0.961±0.063	0.938±0.058	0.940±0.069
1NN_MUL		0.950±0.031	0.923±0.049	0.925±0.047
1NN_OVR		0.950±0.031	0.923±0.049	0.925±0.047
7NN_OVO		0.943±0.062	0.938±0.058	0.928±0.073
MLP		0.936±0.034	0.723±0.158	0.612±0.218

**Table 6:** Performance measures of the considered models for the HFF dataset.

Classifier	Aggregation	ROC AUC	Accuracy	Balanced Accuracy
POM_eGRP	$A_{10}$	0.931±0.029	0.686±0.098	0.649±0.193
POM_eGRP	$A_{5}$	0.930±0.030	0.695±0.093	0.655±0.196
POM_eGRP	$A_{6}$	0.930±0.030	0.695±0.093	0.655±0.196
POM_eGRP	$A_{1}$	0.928±0.026	0.686±0.098	0.649±0.193
POM_eGRP	$A_{2}$	0.928±0.026	0.686±0.098	0.649±0.193
POM_eGRP	$A_{7}$	0.928±0.026	0.686±0.098	0.649±0.193
POM_eGRP	$A_{3}$	0.926±0.034	0.686±0.098	0.648±0.192
POM_eGRP	$A_{9}$	0.926±0.035	0.686±0.098	0.648±0.192
POM_eGRP	$A_{4}$	0.925±0.036	0.686±0.098	0.646±0.192
POM_eGRP	$A_{8}$	0.922±0.036	0.705±0.092	0.659±0.198
POM_ENT	$A_{3}$	0.905±0.041	0.676±0.070	0.643±0.165
POM_ENT	$A_{9}$	0.904±0.041	0.667±0.060	0.637±0.159
POM_ENT	$A_{4}$	0.904±0.040	0.676±0.047	0.643±0.161
POM_ENT	$A_{1}$	0.904±0.043	0.686±0.077	0.651±0.172
POM_ENT	$A_{2}$	0.904±0.043	0.686±0.077	0.651±0.172
POM_ENT	$A_{7}$	0.904±0.043	0.686±0.077	0.651±0.172
POM_ENT	$A_{10}$	0.904±0.044	0.686±0.077	0.651±0.172
POM_ENT	$A_{5}$	0.903±0.044	0.686±0.077	0.651±0.172
POM_ENT	$A_{6}$	0.903±0.042	0.676±0.070	0.643±0.165
POM_ENT	$A_{8}$	0.898±0.040	0.667±0.060	0.636±0.158
POM_ALG	$A_{6}$	0.879±0.033	0.619±0.060	0.546±0.141
POM_ALG	$A_{8}$	0.878±0.037	0.619±0.104	0.608±0.185
POM_ALG	$A_{5}$	0.876±0.034	0.629±0.070	0.552±0.151
POM_ALG	$A_{9}$	0.876±0.035	0.619±0.060	0.546±0.141
POM_ALG	$A_{3}$	0.874±0.039	0.629±0.070	0.552±0.151
POM_ALG	$A_{4}$	0.874±0.037	0.619±0.080	0.546±0.158
POM_ALG	$A_{10}$	0.873±0.030	0.629±0.070	0.552±0.151
POM_ALG	$A_{1}$	0.869±0.030	0.638±0.065	0.619±0.138
POM_ALG	$A_{7}$	0.869±0.031	0.638±0.065	0.619±0.138
POM_ALG	$A_{2}$	0.869±0.030	0.629±0.070	0.552±0.151
RND_FST		0.862±0.063	0.619±0.074	0.477±0.134
BAGGING		0.830±0.042	0.638±0.098	0.684±0.154
SVC		0.822±0.033	0.629±0.036	0.489±0.103
3NN_OVO		0.770±0.126	0.562±0.102	0.515±0.124
7NN_MUL		0.763±0.078	0.629±0.076	0.446±0.057
7NN_OVR		0.756±0.083	0.629±0.070	0.505±0.113
1NN_OVO		0.756±0.155	0.562±0.114	0.572±0.187
5NN_OVR		0.748±0.069	0.610±0.036	0.547±0.137
5NN_MUL		0.748±0.069	0.581±0.056	0.409±0.037
3NN_MUL		0.746±0.062	0.562±0.102	0.515±0.124
3NN_OVR		0.746±0.062	0.552±0.107	0.508±0.134
MLP		0.737±0.107	0.505±0.088	0.413±0.164
5NN_OVO		0.734±0.101	0.590±0.049	0.413±0.035
7NN_OVO		0.732±0.110	0.619±0.080	0.438±0.061
1NN_MUL		0.679±0.140	0.562±0.114	0.572±0.187
1NN_OVR		0.679±0.140	0.562±0.114	0.572±0.187

**Table 7:** Performance measures of the considered models for the SPs dataset.

Classifier	Aggregation	ROC AUC	Accuracy	Balanced Accuracy
BAGGING		0.967±0.027	0.878±0.054	0.878±0.054
POM_ENT	$A_{10}$	0.965±0.021	0.844±0.042	0.844±0.042
POM_ENT	$A_{1}$	0.964±0.021	0.844±0.042	0.844±0.042
POM_ENT	$A_{2}$	0.964±0.021	0.844±0.042	0.844±0.042
POM_ENT	$A_{7}$	0.964±0.021	0.844±0.042	0.844±0.042
POM_eGRP	$A_{8}$	0.963±0.019	0.867±0.057	0.867±0.057
POM_ENT	$A_{5}$	0.963±0.021	0.844±0.042	0.844±0.042
POM_eGRP	$A_{1}$	0.962±0.019	0.867±0.057	0.867±0.057
POM_eGRP	$A_{2}$	0.962±0.019	0.867±0.057	0.867±0.057
POM_eGRP	$A_{4}$	0.962±0.020	0.867±0.057	0.867±0.057
POM_eGRP	$A_{7}$	0.962±0.019	0.867±0.057	0.867±0.057
POM_eGRP	$A_{9}$	0.962±0.020	0.867±0.057	0.867±0.057
POM_eGRP	$A_{3}$	0.961±0.020	0.867±0.057	0.867±0.057
POM_eGRP	$A_{5}$	0.961±0.020	0.867±0.057	0.867±0.057
POM_eGRP	$A_{6}$	0.961±0.020	0.867±0.057	0.867±0.057
POM_eGRP	$A_{10}$	0.961±0.020	0.867±0.057	0.867±0.057
POM_ENT	$A_{3}$	0.961±0.022	0.856±0.027	0.856±0.027
RND_FST		0.960±0.025	0.867±0.057	0.867±0.057
POM_ENT	$A_{4}$	0.959±0.021	0.856±0.027	0.856±0.027
POM_ENT	$A_{6}$	0.959±0.021	0.844±0.042	0.844±0.042
POM_ENT	$A_{8}$	0.959±0.021	0.822±0.042	0.822±0.042
POM_ALG	$A_{1}$	0.958±0.021	0.867±0.044	0.867±0.044
POM_ALG	$A_{2}$	0.958±0.021	0.867±0.044	0.867±0.044
POM_ALG	$A_{7}$	0.958±0.021	0.867±0.044	0.867±0.044
POM_ENT	$A_{9}$	0.958±0.022	0.856±0.027	0.856±0.027
POM_ALG	$A_{10}$	0.957±0.021	0.867±0.067	0.867±0.067
POM_ALG	$A_{5}$	0.955±0.023	0.867±0.067	0.867±0.067
POM_ALG	$A_{6}$	0.955±0.027	0.867±0.067	0.867±0.067
SVC		0.953±0.021	0.867±0.057	0.867±0.057
POM_ALG	$A_{3}$	0.951±0.027	0.844±0.074	0.844±0.074
POM_ALG	$A_{9}$	0.950±0.028	0.856±0.057	0.856±0.057
MLP		0.949±0.030	0.733±0.054	0.733±0.054
POM_ALG	$A_{4}$	0.948±0.030	0.844±0.074	0.844±0.074
POM_ALG	$A_{8}$	0.944±0.031	0.833±0.070	0.833±0.070
3NN_MUL		0.925±0.030	0.778±0.035	0.778±0.035
3NN_OVR		0.925±0.030	0.778±0.035	0.778±0.035
7NN_MUL		0.920±0.022	0.811±0.057	0.811±0.057
7NN_OVR		0.920±0.022	0.811±0.057	0.811±0.057
5NN_MUL		0.911±0.014	0.811±0.027	0.811±0.027
5NN_OVR		0.911±0.014	0.811±0.027	0.811±0.027
7NN_OVO		0.866±0.032	0.811±0.057	0.811±0.057
3NN_OVO		0.852±0.019	0.778±0.035	0.778±0.035
5NN_OVO		0.844±0.023	0.811±0.027	0.811±0.027
1NN_MUL		0.817±0.077	0.756±0.103	0.756±0.103
1NN_OVR		0.817±0.077	0.756±0.103	0.756±0.103
1NN_OVO		0.816±0.070	0.756±0.103	0.756±0.103

**Table 8:** Performance measures of the considered models for the SSh dataset.

Classifier	Aggregation	ROC AUC	Accuracy	Balanced Accuracy
POM_ENT	$A_{9}$	0.815±0.089	0.609±0.099	0.475±0.147
POM_ENT	$A_{6}$	0.814±0.088	0.609±0.078	0.464±0.134
POM_ENT	$A_{7}$	0.813±0.086	0.626±0.081	0.468±0.133
POM_ENT	$A_{10}$	0.812±0.087	0.609±0.067	0.447±0.119
POM_ENT	$A_{5}$	0.812±0.090	0.600±0.070	0.444±0.119
POM_ENT	$A_{1}$	0.811±0.085	0.626±0.081	0.468±0.133
POM_ENT	$A_{2}$	0.809±0.083	0.626±0.081	0.468±0.133
POM_ENT	$A_{4}$	0.808±0.090	0.609±0.099	0.437±0.127
POM_ENT	$A_{3}$	0.807±0.089	0.600±0.084	0.444±0.126
POM_ENT	$A_{8}$	0.806±0.089	0.609±0.078	0.458±0.112
POM_ALG	$A_{5}$	0.796±0.071	0.557±0.097	0.392±0.130
POM_ALG	$A_{10}$	0.794±0.077	0.565±0.099	0.396±0.134
POM_ALG	$A_{7}$	0.793±0.082	0.557±0.097	0.376±0.116
POM_ALG	$A_{1}$	0.791±0.078	0.557±0.097	0.376±0.116
POM_ALG	$A_{6}$	0.790±0.069	0.565±0.099	0.400±0.139
POM_ALG	$A_{2}$	0.789±0.078	0.548±0.098	0.368±0.120
POM_ALG	$A_{4}$	0.783±0.069	0.504±0.128	0.358±0.134
POM_eGRP	$A_{4}$	0.782±0.107	0.583±0.085	0.446±0.139
POM_eGRP	$A_{9}$	0.782±0.106	0.583±0.071	0.446±0.134
POM_ALG	$A_{9}$	0.780±0.072	0.522±0.091	0.361±0.122
POM_ALG	$A_{8}$	0.778±0.083	0.478±0.082	0.397±0.113
POM_ALG	$A_{3}$	0.778±0.073	0.565±0.099	0.410±0.135
POM_eGRP	$A_{6}$	0.777±0.105	0.591±0.076	0.454±0.142
POM_eGRP	$A_{8}$	0.777±0.118	0.591±0.090	0.459±0.128
POM_eGRP	$A_{3}$	0.776±0.107	0.591±0.071	0.450±0.133
POM_eGRP	$A_{5}$	0.776±0.105	0.591±0.076	0.454±0.142
POM_eGRP	$A_{1}$	0.769±0.099	0.583±0.081	0.446±0.144
POM_eGRP	$A_{10}$	0.769±0.097	0.591±0.076	0.454±0.142
POM_eGRP	$A_{7}$	0.768±0.101	0.583±0.081	0.446±0.144
POM_eGRP	$A_{2}$	0.766±0.098	0.583±0.081	0.446±0.144
SVC		0.761±0.074	0.522±0.027	0.300±0.063
RND_FST		0.737±0.069	0.565±0.055	0.383±0.106
7NN_MUL		0.720±0.091	0.539±0.071	0.464±0.139
3NN_OVR		0.714±0.081	0.548±0.081	0.418±0.078
3NN_MUL		0.714±0.081	0.530±0.118	0.521±0.196
7NN_OVR		0.711±0.091	0.539±0.044	0.412±0.075
7NN_OVO		0.706±0.075	0.574±0.051	0.436±0.086
5NN_OVR		0.705±0.080	0.539±0.059	0.415±0.066
5NN_MUL		0.705±0.080	0.478±0.103	0.424±0.130
5NN_OVO		0.705±0.099	0.557±0.070	0.449±0.124
BAGGING		0.704±0.037	0.530±0.075	0.443±0.115
3NN_OVO		0.699±0.067	0.487±0.075	0.393±0.089
1NN_OVO		0.689±0.070	0.426±0.104	0.363±0.134
MLP		0.662±0.090	0.339±0.104	0.241±0.067
1NN_MUL		0.602±0.084	0.426±0.104	0.363±0.134
1NN_OVR		0.602±0.084	0.426±0.104	0.363±0.134

**Table 9:** Kruskal–Wallis and Dunn-Bonferroni test results, part I.

ALL				BTu			
*p*-Value	<0.0001			*p*-Value	0.0049		
	POM_ALG	POM_ENT	POM_eGRP		POM_ALG	POM_ENT	POM_eGRP
POM_ALG	1.0000	0.1179	**<0.0001**	POM_ALG	1.0000	0.4864	**0.0034**
POM_ENT	0.1179	1.0000	**0.0160**	POM_ENT	0.4864	1.0000	0.1906
POM_eGRP	**<0.0001**	**0.0160**	1.0000	POM_eGRP	**0.0034**	0.1906	1.0000

**Table 10:** Kruskal–Wallis and Dunn-Bonferroni test results, part II.

HeC				HFF			
*p*-Value	<0.0001			*p*-Value	<0.0001		
	POM_ALG	POM_ENT	POM_eGRP		POM_ALG	POM_ENT	POM_eGRP
POM_ALG	1.0000	**0.0001**	**0.0007**	POM_ALG	1.0000	**0.0328**	**<0.0001**
POM_ENT	**0.0001**	1.0000	1.0000	POM_ENT	**0.0328**	1.0000	**0.0328**
POM_eGRP	**0.0007**	1.0000	1.0000	POM_eGRP	**<0.0001**	**0.0328**	1.0000

**Table 11:** Kruskal–Wallis and Dunn-Bonferroni test results, part III.

SSh				SPs			
*p*-Value	<0.0001			*p*-Value	<0.0001		
	POM_ALG	POM_ENT	POM_eGRP		POM_ALG	POM_ENT	POM_eGRP
POM_ALG	1.0000	**0.0213**	0.0762	POM_ALG	1.0000	**0.0004**	**0.0005**
POM_ENT	**0.0213**	1.0000	**<0.0001**	POM_ENT	**0.0004**	1.0000	1.0000
POM_eGRP	0.0762	**<0.0001**	1.0000	POM_eGRP	**0.0005**	1.0000	1.0000

## Data Availability

The data utilized in this study can be made accessible upon request from the corresponding author, who will provide it in accordance with their current status.
